# Molecular insights into floral scent biosynthesis in *Rosa laevigata* through transcriptomic and metabolomic analyses

**DOI:** 10.3389/fpls.2025.1599758

**Published:** 2025-06-23

**Authors:** Jian Ru, Wen-Bin Ju, Liang-Ying Li, Heng-Ning Deng, Zhen-Long Liang, Zhong-Yu Tang, Jia Miao, Cheng Zhang, Xin-Fen Gao

**Affiliations:** ^1^ Mountain Ecological Restoration and Biodiversity Conservation Key Laboratory of Sichuan Province, Chengdu Institute of Biology, Chinese Academy of Sciences, Chengdu, Sichuan, China; ^2^ Key Laboratory for Regional Plants Conservation and Ecological Restoration of Northeast Jiangxi, College of Life Science, Shangrao Normal University, Shangrao, Jiangxi, China; ^3^ University of Chinese Academy of Sciences, Beijing, China; ^4^ Yunnan Key Laboratory of Sustainable Utilization Research on Rubber Tree, the Center of Rubber Research, Yunnan Institute of Tropical Crops, Xishuangbanna, Yunnan, China

**Keywords:** *Rosa laevigata* Michx., floral scent, metabolome, transcriptome, transcription factors

## Abstract

*Rosa laevigata* Michx., a member of the genus *Rosa* in Rosaceae, has large, pure white flowers with a pleasant floral scent, making it a valuable ornamental and aromatic plant. However, the composition and dynamic changes in the abundance of its volatile organic compounds (VOCs) at different developmental stages, as well as the molecular mechanisms regulating floral scent biosynthesis, remain unclear. In this study, we conducted metabolomic and transcriptomic analyses to investigate the composition and abundance changes of VOCs in *R. laevigata* flowers at three developmental stages. Additionally, we identified key structural genes involved in the floral scent biosynthesis pathways. The results showed that a total of 330 VOCs were identified during the three developmental stages, of which 192 were differential volatile organic compounds (DVOCs), mainly benzenoids/phenylpropanoids and esters. Transcriptomic analysis further identified 8,585 differentially expressed genes, of which 67 were key structural genes related to floral scent biosynthesis. The regulatory network of transcription factors and structural genes revealed that 20 transcription factors were highly associated with floral scent biosynthesis. These findings provide a theoretical basis for the molecular breeding of fragrant germplasm in *R. laevigata* and contribute to the development of its aromatic industry.

## 1 Introduction

*Rosa laevigata* Michx., belonging to the genus *Rosa* in Rosaceae, is a perennial evergreen climbing shrub distributed in Zhejiang, Guangdong, Jiangxi, Sichuan, and Yunnan provinces (Yu et al., 1985). *R. laevigata* is a renowned traditional Chinese medicinal plant known for its dual use in medicine and food (Li et al., 2021; Quan et al., 2022). Additionally, *R. laevigata* is highly adaptable and characterized by large, pure white flowers with a pleasant fragrance, making it a valuable ornamental aromatic plant often used in garden landscaping, landscape design, or as a hedge plant (Zhang et al., 2019; Jiao, 2022).

Floral scent is composed of various lipophilic, low molecular weight (100–250 Da), and low boiling point VOCs that are released into the surrounding air through the floral organ of the plant. These VOCs are classified into seven major categories: aliphatics, benzenoids/phenylpropanoids, terpenoids, C5-branched compounds, heterocyclic compounds, nitrogen-containing, and sulfur-containing compounds (Dudareva et al., 1999; Knudsen et al., 2006). Floral scent is often believed to attract pollinators while also protect the plant from herbivores and pathogens (Muhlemann et al., 2014; Loreto and D’Auria, 2022). In ornamental flowers, floral scent is one of the key traits for evaluating the ornamental value of blooming plants and significantly influences consumer preferences (In et al., 2021). Additionally, floral scent is widely used in industries such as perfumery, cosmetics, flavorings, and pharmaceuticals, holding significant economic value (Dudareva and Pichersky, 2000; Mostafa et al., 2022). Research on the floral scent of *R. laevigata* focuses on the types and abundances of VOCs present in whole flowers or petals at the full blooming stage. Wang (2019) identified 10 VOCs in the petals of *R. laevigata*, with pentadecanone, benzyl alcohol, and hexyl decanoate being the most abundant. Jiao (2022) identified 31 VOCs in the whole flowers, with eicosane, heptadecene, and benzyl alcohol as the primary volatiles. However, the composition and abundance changes of VOCs in the whole flower of *R. laevigata* at different developmental stages, as well as the molecular mechanisms underlying floral scent biosynthesis, have not yet been reported.

In recent years, combined transcriptomic and metabolomic analyses have been widely used in the study of floral scent (Wang et al., 2022; Zhu et al., 2022; Ke et al., 2023). In this study, headspace solid-phase microextraction-gas chromatography-mass spectrometry (HS-SPME-GC-MS) was employed to detect VOCs in the flowers of *R. laevigata* at three different developmental stages. Additionally, RNA-seq was used to investigate the expression patterns of genes related to floral scent biosynthesis. Our objectives were to elucidate (1) the dynamic changes in the composition and relative abundances of VOCs in flowers of *R. laevigata* at three developmental stages; (2) the biosynthetic pathways associated with floral scent formation; and (3) the expression patterns of key genes regulating floral scent biosynthesis. These findings contribute to the understanding of the molecular mechanisms of *R. laevigata* floral scent biosynthesis, provide valuable data for the molecular breeding of aromatic *R. laevigata*, and promote the development of the *R. laevigata* aromatic industry.

## 2 Materials and methods

### 2.1 Plant materials

**Figure 1:**
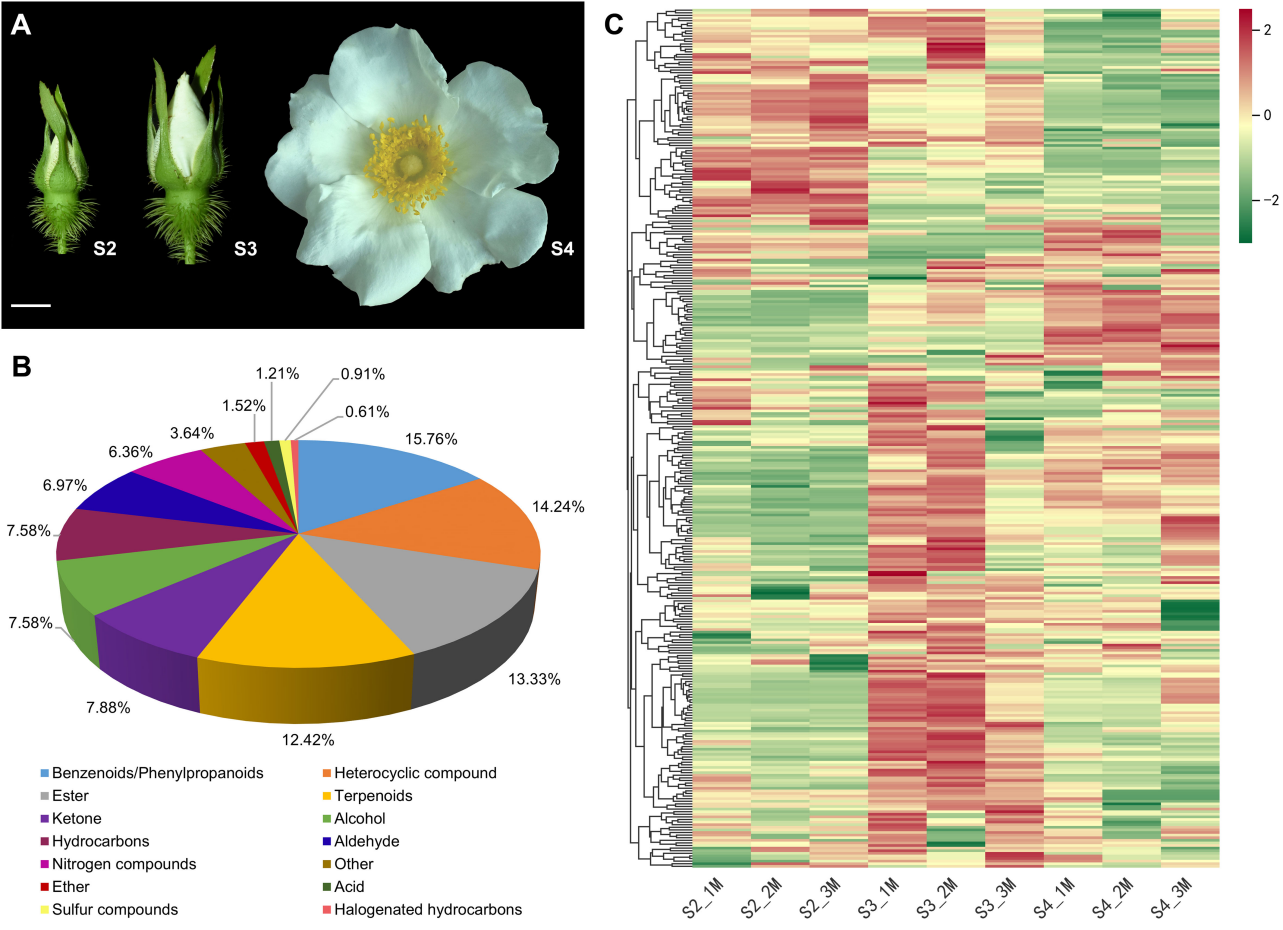
Phenotypes and metabolome of *R. laevigata* flowers at three developmental stages. **(A)** Phenotypes of flowers at the bud stage (S2), initial blooming stage (S3), and full blooming stage (S4). Scale bar = 1 cm. **(B)** Classification of VOCs. **(C)** Hierarchical clustering heatmap of 330 VOCs.

*R. laevigata* was cultivated in the *Rosa* Germplasm Resource Garden at the Chengdu Institute of Biology, Chinese Academy of Sciences, Chengdu, China. To investigate the VOCs composition and molecular mechanisms of floral scent biosynthesis of *R. laevigata* flowers at three developmental stages, flowers were collected on sunny mornings in April 2023. The developmental stages were the bud stage (sepals closed, corolla slightly visible between sepals, S2), the initial blooming stage (sepals open, exposing the pinched corolla, S3), and the full blooming stage (corolla fully open, stamens and pistils exposed, S4) (**Figure 1A**). The collected samples were wrapped in aluminum foil, rapidly frozen in liquid nitrogen, and then stored at -80°C until further analysis. Six biological replicates were collected at each developmental stage, of which three were used for VOCs analysis and the remaining three for RNA-seq.

### 2.2 Analysis of VOCs with HS-SPME-GC-MS

VOCs from the flowers were collected using HS-SPME. A 50/30 µm DVB/CAR/PDMS fiber was exposed to the headspace for 30 min and then desorbed at 250°C for 4 min. The collected VOCs were analyzed by GC-MS using an Agilent 7890 gas chromatograph system coupled with an Agilent 5977B mass spectrometer. The system utilized a DB-Wax capillary column (30 m×250 μm×0.25 μm). High-purity helium was used as the carrier gas, the front inlet purge flow was 3 mL/min, and the gas flow rate through the column was 1 mL/min. Injected in splitless mode, solvent delay of 2.13 min. The initial temperature was kept at 40°C for 4 min, then raised to 245°C at a rate of 5°C/min, kept for 7 min. The injection, transfer line, ion source and quad temperatures were 250°C, 250°C, 230°C and 150°C, respectively. The electron ionization mode at 70 eV. The mass spectrometry data were acquired in scan mode with the m/z range of 20-400. Chroma TOF 4.3X software of LECO Corporation and the Nist database were used for raw peaks exacting, the data baselines filtering and calibration of the baseline, peak alignment, deconvolution analysis, peak identification, integration and spectrum match of the peak area (Kind et al., 2009).

### 2.3 RNA extraction, cDNA library construction, sequencing, and transcript quantification

Total RNA of 9 samples were isolated using the Trizol Reagent (Invitrogen Life Technologies), after which the purity and integrity were determined using a NanoDrop spectrophotometer (Thermo Fisher Scientific, Massachusetts, USA) and Agilent 2100 Bioanalyzer (Agilent Technologies, California, USA), respectively. Sequencing libraries were generated using NEBNext Ultra II RNA Library Prep Kit for Illumina (NEB, Ipswich, Massachusetts, USA) according to the manufacturer’s protocol. To select cDNA fragments of the preferred 400–500 bp in length, the library fragments were purified using the AMPure XP system (Beckman Coulter, Beverly, CA, USA). DNA fragments with ligated adaptor molecules on both ends were selectively enriched using Illumina PCR Primer Cocktail in a 15 cycle PCR reaction. Products were purified (AMPure XP system) and quantified using the Agilent high sensitivity DNA assay on an Agilent Bioanalyzer 2100 system. The sequencing library was then sequenced on Illumina NovaSeq X Plus platform Shanghai Personal Biotechnology Cp. Ltd. The sequencing data have been deposited into CNGB Sequence Archive (CNSA) (Guo et al., 2020) of China National GeneBank DataBase (CNGBdb) (Chen et al., 2020) with accession number CNP0005764.

The raw data were filtered, resulting in clean reads (Chen et al., 2018), which were mapped to reference genome (https://plants.ensembl.org/Rosa_chinensis/Info/Index) using HISAT2 (Kim et al., 2015). HTSeq was used to obtain the read count values mapped to each gene, representing the raw expression levels, which were then normalized using FPKM (Mortazavi et al., 2008; Anders et al., 2015).

### 2.4 Data analysis of differential VOCs and differentially expressed genes

Metabolomic data were analyzed using SIMCA software (V16.0.2, Sartorius Stedim Data Analytics AB, Umea, Sweden) for principal component analysis (PCA) and orthogonal projections to latent structures discriminant analysis (OPLS-DA). To identify differential VOCs (DVOCs) between different comparison groups, we used a threshold of variable importance for projection (VIP) > 1 and P-value < 0.05. All DVOCs were standardized using z-score normalization, and K-means clustering was performed using the cluster (v2.1.0) and fpc (v2.2-9) packages in R to group metabolites with similar accumulation trends across developmental stages.

Differential expression analysis for transcriptomic data between different comparison groups was conducted using DESeq2 (Love et al., 2014). Differentially expressed genes (DEGs) were selected based on the criteria of |log2Fold Change| > 1 and P-value < 0.05. Gene Ontology (GO) enrichment analysis and KEGG pathway enrichment analysis was conducted using clusterProfiler (Yu et al., 2012). The Pearson correlation coefficients between DVOCs and DEGs, as well as transcription factors (TFs) and DEGs, were calculated using R. Correlation networks were visualized using Cytoscape (version 3.10.2).

### 2.5 qRT-PCR validation

Nine genes related to floral scent biosynthesis were selected for qRT-PCR validation. RNA was reverse-transcribed into cDNA using the PrimeScript™ 1st strand cDNA Synthesis Kit. The qRT-PCR experiments were conducted on a LightCycler 480II, 384 real-time fluorescence quantitative detection system. The GAPDH gene was used as an internal reference gene (Wang, 2019), with three biological replicates for each analysis. Relative expression levels were calculated using the 2^-ΔΔCt^ method (Livak and Schmittgen, 2001). The primer sequences are listed in **Supplementary Table S1**.

## 3 Results

### 3.1 VOCs of *R. laevigata* flowers

The characteristics of VOCs in *R. laevigata* flowers at three developmental stages (**Figure 1A**) were analyzed using HS-SPME-GC-MS. A total of 330 VOCs were identified during three developmental stages, and 330, 325, and 325 VOCs identified at stages S2, S3, and S4, respectively (**Supplementary Table S2**). These VOCs were categorized into 14 groups (**Figure 1B**), including benzenoids/phenylpropanoids (52, 15.76%), heterocyclic compounds (47, 14.24%), esters (44, 13.33%), terpenoids (41, 12.42%), ketones (26, 7.88%), alcohols (25, 7.58%), hydrocarbons (25, 7.58%), aldehydes (23, 6.97%), nitrogen-containing compounds (21, 6.36%), ethers (5, 1.52%), acids (4, 1.21%), sulfur-containing compounds (3, 0.91%), halogenated hydrocarbons (2, 0.61%), and others (12, 3.64%). Among these, benzenoids/phenylpropanoids, heterocyclic compounds, esters, and terpenoids accounted for 55.76% of the total VOCs (**Figure 1B**; **Supplementary Table S2**). Hierarchical clustering analysis indicated that the relative abundance of VOCs showed significant changes during the three developmental stages, with most VOCs exhibiting higher relative abundance at the S3 stage (**Figure 1C**). PCA results indicated that samples from the three developmental stages could be distinctly separated (**Supplementary Figure S1**).

### 3.2 Differential volatile organic compounds

**Figure 2:**
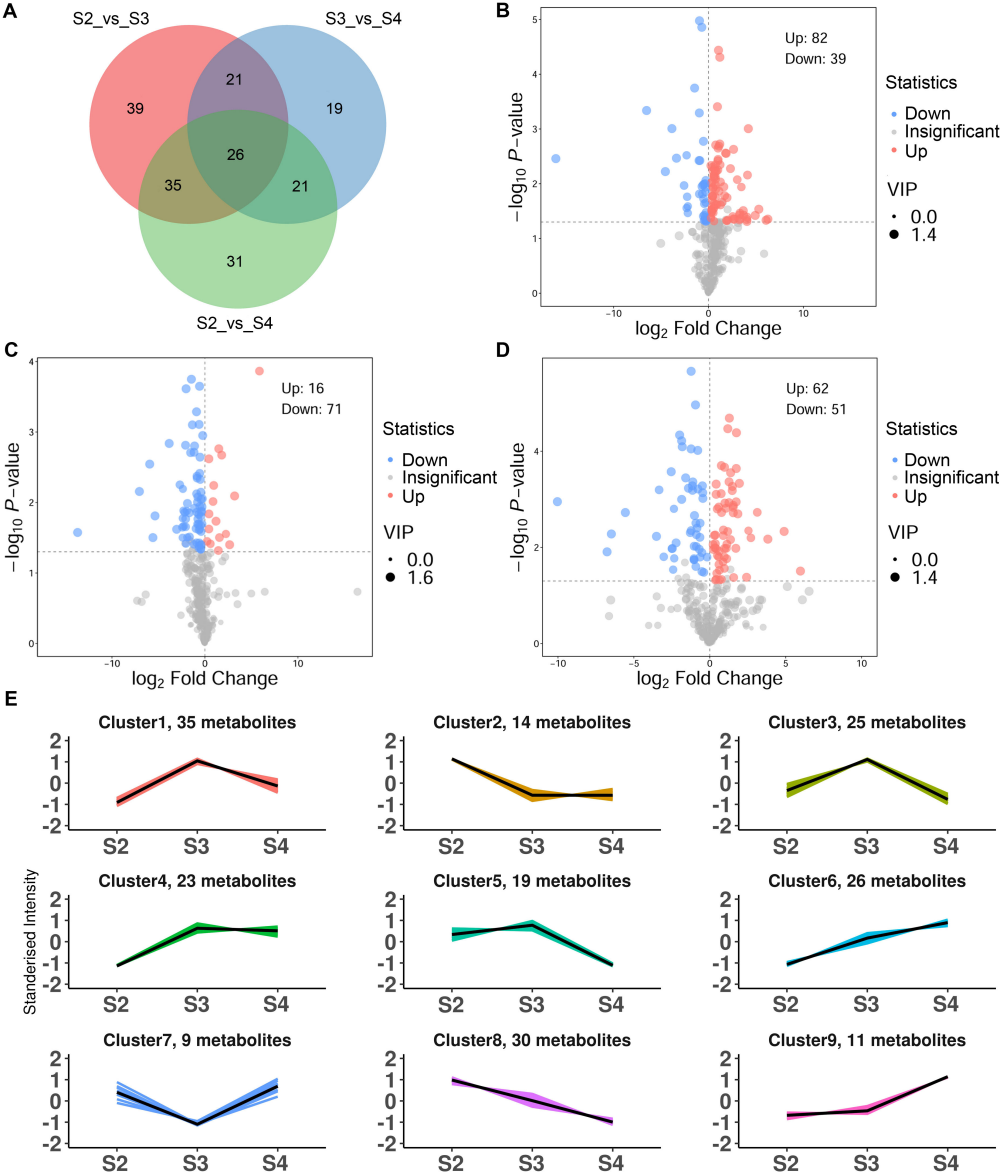
Characteristics of DVOCs of *R. laevigata* flowers. **(A)** Venn diagram. **(B-D)** Volcano plot in S2 vs. S3, S3 vs. S4, and S2 vs. S4, respectively. **(E)** K-means analysis.

To further elucidate the differences in VOCs at three developmental stages, OPLS-DA was employed to screen for DVOCs in three comparison groups (S2 vs. S3, S3 vs. S4 and S2 vs. S4). The R^2^Y and Q^2^ values for each OPLS-DA model exceeded 0.9, and their associated P-values were both below 0.05, indicating that the OPLS-DA models effectively explained the differences in VOCs during the developmental stages (**Supplementary Figures S1B–D**). A total of 192 DVOCs were identified among the three comparison groups, of which 26 were shared DVOCs, and 39, 19, and 31 were unique to S2 vs. S3, S3 vs. S4, and S2 vs. S4, respectively (**Figure 2A**; **Supplementary Table S3**). Among the DVOCs, benzenoids/phenylpropanoids (28, 14.58%) and esters (28, 14.58%) were the most abundant, followed by terpenoids (27, 14.06%), heterocyclic compounds (24, 12.50%), and aldehydes (17, 8.85%). In S2 vs. S3, 121 DVOCs were identified, of which 82 showed increased abundance and 39 showed decreased abundance in S3 (**Figure 2B**). In S3 vs. S4, 87 DVOCs were identified, of which 16 showed increased abundance and 71 showed decreased abundance in S4 (**Figure 2C**). In S2 vs. S4, 113 DVOCs were identified, of which 62 showed increased abundance and 51 showed decreased abundance in S4 (**Figure 2D**).

K-means clustering partitioned the 192 DVOCs into nine clusters (**Figure 2E**; **Supplementary Table S3**), containing 35, 14, 25, 23, 19, 26, 9, 30 and 11 compounds in clusters 1 to 9, respectively. Clusters 1, 3, 4 and 5 included 102 DVOCs whose relative abundances increased from S2, peaked at S3, and declined at S4, with benzenoids/phenylpropanoids (19, 18.63%) and monoterpenoids (16, 15.69%) being the dominant classes, followed by hydrocarbons, ketones, esters, heterocyclic compounds, aldehydes, sesquiterpenoids and alcohols. Clusters 2 and 8 comprised 44 DVOCs that showed a continuous decrease from S2 to S4. Heterocyclic compounds (9, 20.45 %) and aldehydes (7, 15.91 %) were most abundant classes in these clusters, followed by esters (6), nitrogen−containing compounds (6), alcohols (4), monoterpenoids (4), as well as smaller numbers of halogenated hydrocarbons, hydrocarbons, ketones and a single acid. Cluster 7 consisted of 9 DVOCs. Heterocyclic compounds (3, 33.33%) were the largest class, followed by esters (2, 22.22%), with one compound each from the ketone, sesquiterpenoid, acid and hydrocarbon categories. The abundances of these DVOCs declined from S2 to a minimum at S3 and increased again at S4. Clusters 6 and 9, which exhibited a gradually increasing abundance trend from S2 to S4, 37 DVOCs were dominated by esters (10, 27.03%) and benzenoids/phenylpropanoids (9, 24.32%), followed by alcohols (5), heterocyclic compounds (3), nitrogen-containing compounds (3), aldehydes (3), ketones (2), terpenoids (1) and other (1).

### 3.3 Transcriptome analysis of *R. laevigata* flowers at three developmental stages

To further investigate the molecular mechanisms underlying the floral scent biosynthesis in *R. laevigata* flowers, we constructed and sequenced nine cDNA libraries from total RNA extracted at the S2, S3, and S4 stages. A total of 147,070,232, 150,761,094, and 130,587,874 clean reads were obtained from the S2, S3, and S4 samples, respectively. The Q20 and Q30 ranged from 96.68% to 96.97% and 94.09% to 94.6%, respectively, with GC content between 45.66% and 45.83%. On average, 73.33% of the clean reads were aligned to the reference genome (**Supplementary Table S4**). PCA and correlation analysis indicated reproducibility among samples from the same developmental stage and distinct differences among stages, confirming the suitability of transcriptome data for subsequent analyses (**Supplementary Figure S2**).

**Figure 3:**
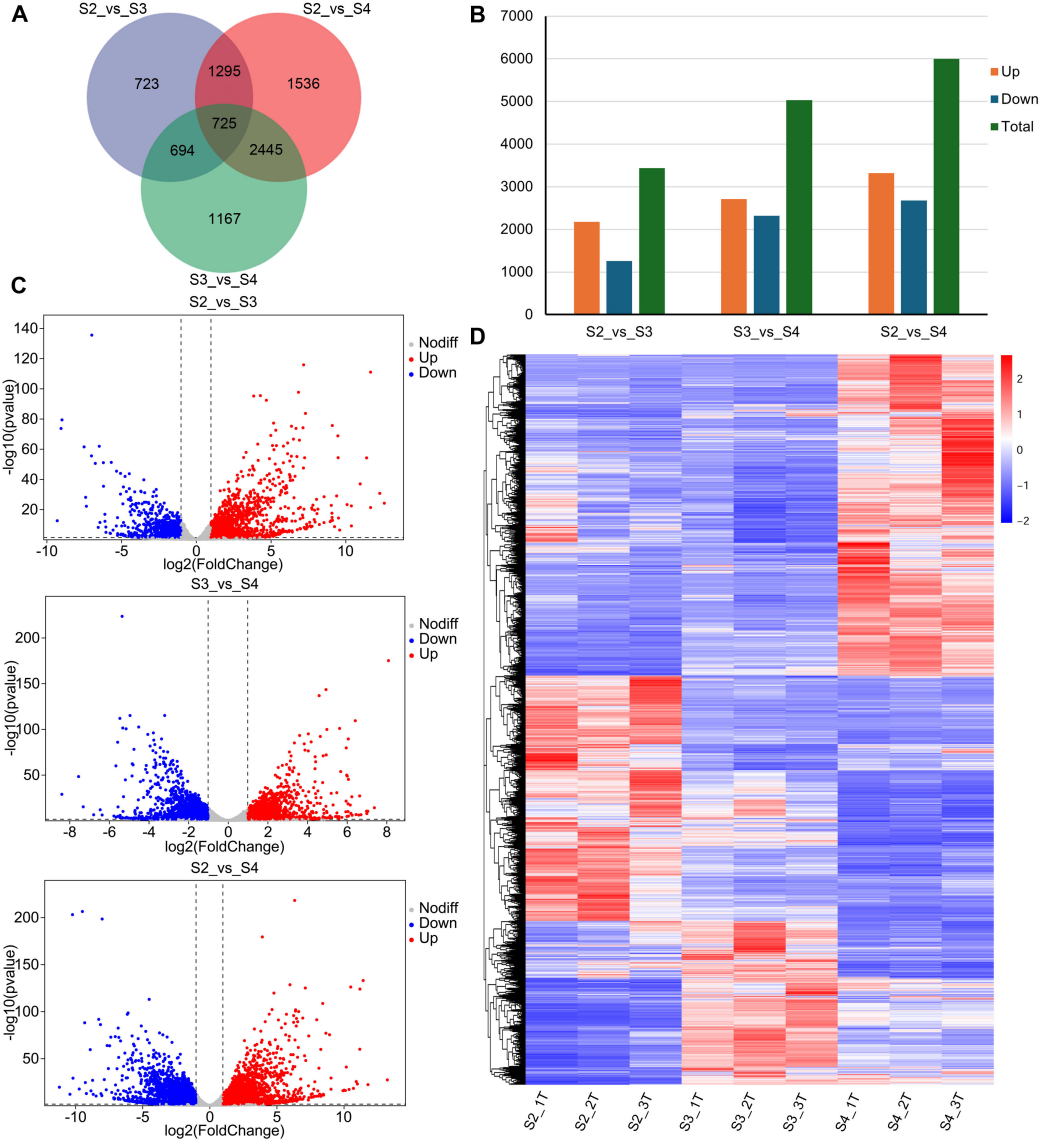
Analysis of DEGs of *R. laevigata* flowers at three developmental stages. **(A)** Venn diagram of DEGs among three comparison groups. **(B)** The number of DEGs in three comparison groups. **(C)** Volcano plot of DEGs in three comparison groups. **(D)** Hierarchical clustering heatmap of DEGs.

A total of 8,585 DEGs were identified, of which 725 were shared among the three comparison groups (S2 vs. S3, S2 vs. S4, and S3 vs. S4) (**Figure 3A**; **Supplementary Table S5**). In S2 vs. S3, 3,437 DEGs were identified, of which 2,180 were upregulated and 1,257 were downregulated in S3 (**Figures 3B, C**). In S3 vs. S4, 5,031 DEGs were identified, with 2,711 upregulated and 2,320 downregulated in S4 (**Figures 3B, C**). In S2 vs. S4, 6,001 DEGs were identified, with 3,322 upregulated and 2,679 downregulated in S4 (**Figures 3B, C**). As the *R. laevigata* flowers developed, the number of DEGs was greater in S3 vs. S4 than in S2 vs. S3. Hierarchical clustering analysis categorized the DEGs into three major clusters (**Figure 3D**).

The GO enrichment analysis results showed that in the biological process (BP) category, the three comparison groups were primarily enriched in the carbohydrate metabolic process (GO:0005975). In the molecular function (MF) category, the comparison groups were primarily enriched in transporter activity (GO:0005215). In the cellular component (CC) category, S2 vs. S3 and S2 vs. S4 were mainly enriched in the extracellular region (GO:0005576), while S3 vs. S4 was primarily enriched in the photosystem (GO:0009521) (**Supplementary Figure S3**).

**Figure 4:**
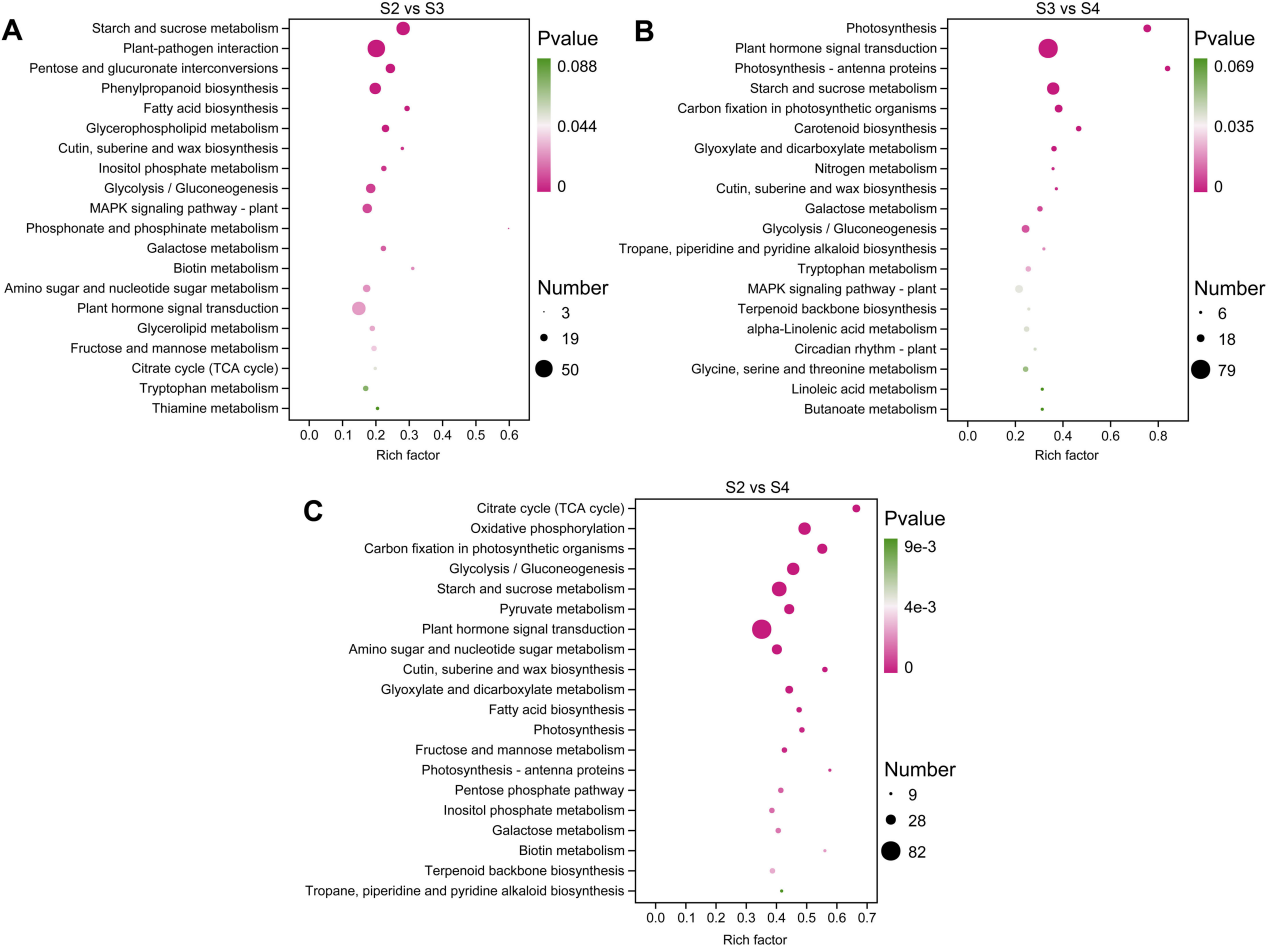
The top 20 significantly enriched KEGG pathways in three comparison groups. **(A)** S2 vs. S3. **(B)** S3 vs. S4. **(C)** S2 vs. S4.

KEGG pathway enrichment analysis was used to functionally classify the DEGs and identify metabolic pathways related to floral scent biosynthesis. In S2 vs. S3, S2 vs. S4, and S3 vs. S4 comparisons, 112, 120, and 115 pathways were enriched, respectively (**Supplementary Tables S6**–**S8**). The significantly enriched KEGG pathways are shown in **Figures 4A–C**, among which the pathways related to floral scent biosynthesis include phenylpropanoid biosynthesis (ko00940), terpenoid backbone biosynthesis (ko00900), and α-linolenic acid metabolism (ko00592). Additionally, other pathways related to floral scent biosynthesis were also enriched, including the downstream pathways of terpenoid biosynthesis, such as sesquiterpenoid and triterpenoid biosynthesis (ko00909) and diterpenoid biosynthesis (ko00904) (**Supplementary Tables S6**–**S8**).

### 3.4 DEGs related to phenylpropanoid biosynthesis

**Figure 5:**
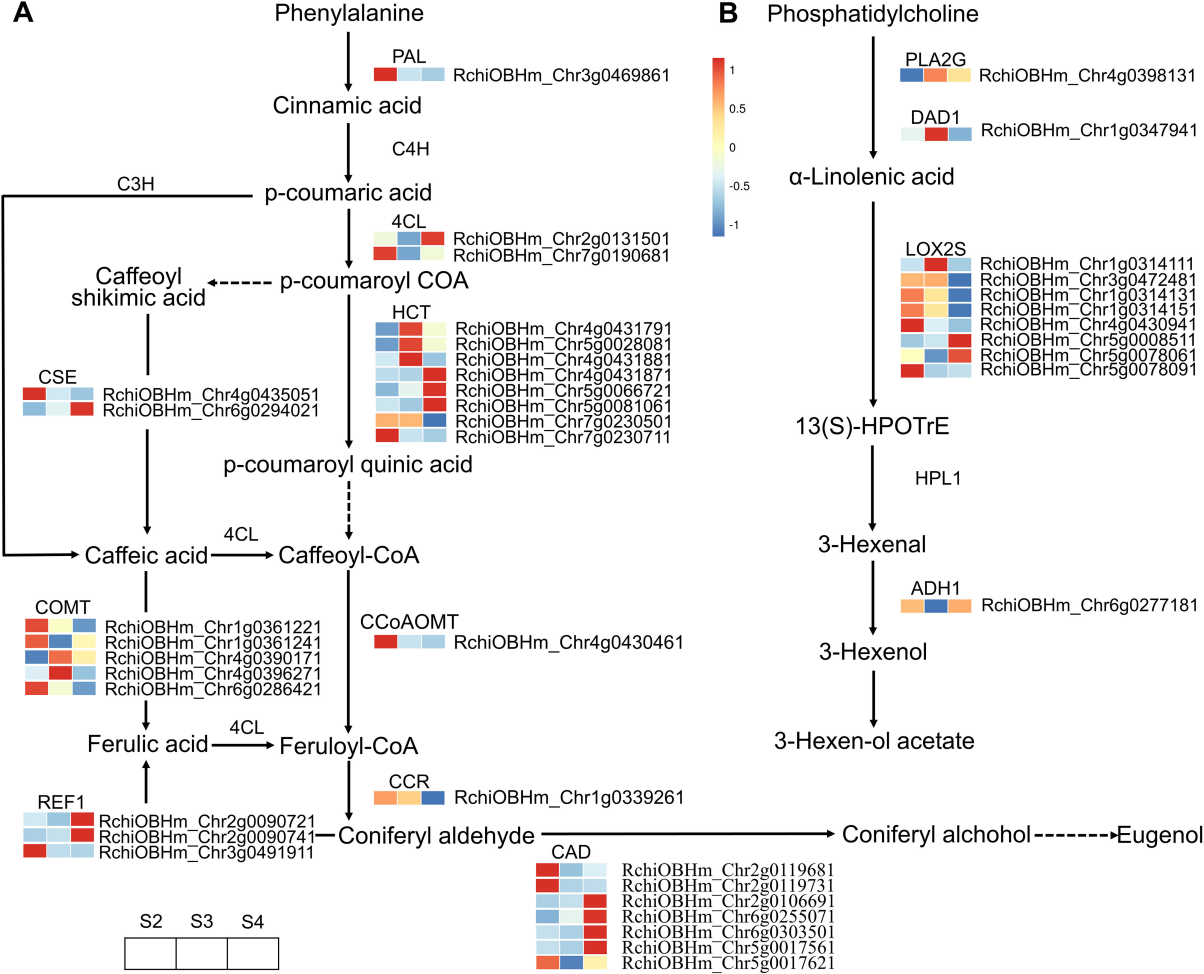
Expression profiles of genes related to phenylpropanoid biosynthesis and α-linolenic acid metabolism in *R. laevigata* flowers at three developmental stages. **(A)** Phenylpropanoid biosynthesis. **(B)** α-Linolenic acid metabolism.

A total of 57 DEGS were enriched in the phenylpropanoid biosynthesis pathway, of which 30 were related to floral scent biosynthesis (**Figure 5A**; **Supplementary Table S9**). These DEGs exhibited varying expression patterns. Genes continuously upregulated from S2 to S4 included one gene (RchiOBHm_Chr5g0066721) encoding shikimate O-hydroxycinnamoyltransferase (HCT), one gene (RchiOBHm_Chr6g0294021) encoding caffeoylshikimate esterase (CSE), one gene (RchiOBHm_Chr2g0090741) encoding coniferyl-aldehyde dehydrogenase (REF1), and two genes (RchiOBHm_Chr2g0106691 and RchiOBHm_Chr6g0255071) encoding cinnamyl-alcohol dehydrogenase (CAD). The expression trends of these genes were consistent with the relative abundance changes of the identified VOCs, including eugenol, 3-phenylpropanol, benzyl chloride, 1H-inden-1-ol, 2,3-dihydro-, phenol, 4-(2-propenyl)-, and phenol, 3-methyl-. However, three genes (RchiOBHm_Chr4g0431791, RchiOBHm_Chr4g0431881, RchiOBHm_Chr5g0028081) encoding HCT and two genes (RchiOBHm_Chr4g0390171 and RchiOBHm_Chr4g0396271) encoding caffeic acid 3-O-methyltransferase (COMT) showed peak expression levels at S3, followed by a gradual decrease at S4. The relative abundance of VOCs such as 2-propen-1-ol, 3-phenyl-, (E)-, phenol, 2-methoxy-, styrene, and benzene, 1-ethynyl-4-methyl- exhibited a trend of initially increasing and then decreasing.

### 3.5 DEGs related to terpenoid biosynthesis

**Figure 6:**
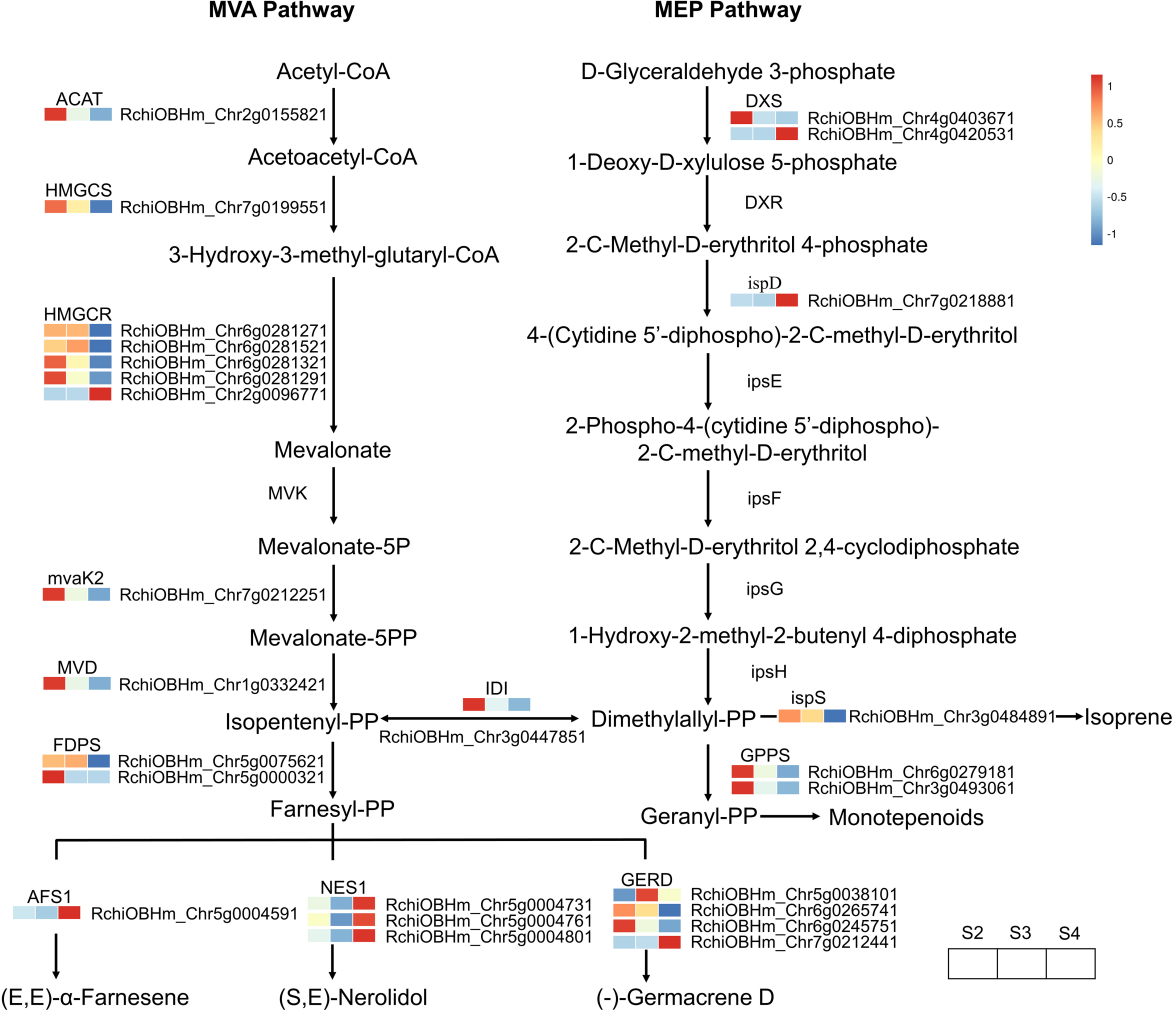
Expression profiles of genes related to terpenoid biosynthesis in *R. laevigata* flowers at three developmental stages.

A total of 26 DEGs genes were enriched in the terpenoid biosynthesis pathway (**Supplementary Table S10**). The biosynthesis of upstream precursor substances of terpenoids occurs through the terpenoid backbone pathway (ko00900), which includes the mevalonate pathway (MVA) and the methylerythritol 4-phosphate pathway (MEP) (**Figure 6**). Within these two pathways, only one gene (RchiOBHm_Chr2g0096771) encoding hydroxymethylglutaryl-CoA reductase (HMGCR) was gradually upregulated during the development of *R. laevigata* flowers. Conversely, one gene (RchiOBHm_Chr2g0155821) encoding acetyl-CoA C-acetyltransferase (ACAT), one gene (RchiOBHm_Chr7g0199551) encoding hydroxymethylglutaryl-CoA synthase (HMGCS), three genes (RchiOBHm_Chr6g0281271, RchiOBHm_Chr6g0281321, RchiOBHm_ Chr6g0281291) encoding hydroxymethylglutaryl-CoA reductase (HMGCR), one gene (RchiOBHm_Chr7g0212251) encoding phosphomevalonate kinase (mvaK2), one gene (RchiOBHm_Chr1g0332421) encoding diphosphomevalonate decarboxylase (MVD), and one gene (RchiOBHm_Chr4g0403671) encoding 1-deoxy-D-xylulose-5-phosphate synthase (DXS) were highly expressed at S2; however, their expression levels gradually decreased during flower development. Compared to the genes regulating upstream precursor biosynthesis, 10 DEGs were related to sesquiterpenoid biosynthesis, and 2 DEGs were related to monoterpenoid biosynthesis in the downstream pathways. In the sesquiterpenoid biosynthesis, one gene (RchiOBHm_Chr5g0075621) encoding farnesyl diphosphate synthase (FDPS) and one gene (RchiOBHm_Chr5g0038101) encoding (-)-germacrene D synthase were highly expressed at S3 and downregulated at S4. Conversely, one gene (RchiOBHm_Chr5g0004591) encoding alpha-farnesene synthase (AFS1) and three genes (RchiOBHm_Chr5g0004731, RchiOBHm_Chr5g0004761, RchiOBHm_ Chr5g0004801) encoding (3S,6E)-nerolidol synthase (NES1) were downregulated from S2 to S3 and then upregulated at S4. In the monoterpenoid biosynthesis pathway, the expression levels of two genes encoding geranylgeranyl diphosphate synthase (GGPS) gradually decreased during flower development.

### 3.6 DEGs related to fatty acid derivative metabolism pathway

In the fatty acid derivative metabolism pathway, 11 DEGs were enriched, of which 8 genes encoded lipoxygenase (LOX2S) (**Figure 5B**; **Supplementary Table S11**). However, these DEGs exhibited various expression patterns during the development of *R. laevigata* flowers. The expression level of RchiOBHm_Chr5g0008511 gradually upregulated, while RchiOBHm_Chr1g0314131, RchiOBHm_Chr1g0314151, and RchiOBHm_Chr4g0430941 gradually decreased. RchiOBHm_Chr1g0314111 and RchiOBHm_Chr3g0472481 showed higher expression levels at S3 compared to S2 and S4. Conversely, RchiOBHm_Chr5g0078061 and RchiOBHm_Chr5g0078091 were downregulated from S2 to S3, and then upregulated from S3 to S4. Additionally, one gene (RchiOBHm_Chr1g0347941) encoding phospholipase A1 (DAD1) and one gene (RchiOBHm_Chr4g0398131) encoding secretory phospholipase A2 (PLA2G) exhibited an initial upregulation followed by a downregulation trend. Conversely, one gene (RchiOBHm_Chr6g0277181) encoding alcohol dehydrogenase (ADH1) exhibited an initial downregulation followed by upregulation.

### 3.7 Combined analysis of metabolome and transcriptome

To comprehensively elucidate the regulatory mechanisms associated with the biosynthesis of floral scent in *R. laevigata*, we performed an integrated analysis of metabolomic and transcriptomic data, focusing on both metabolite-gene and TF-gene associations.

From the 192 DVOCs identified, 34 volatiles previously reported to possess characteristic floral scent properties were selected for correlation analysis. These volatiles were correlated with 67 structural genes involved in phenylpropanoid biosynthesis, terpenoid biosynthesis, and fatty acid derivative metabolism pathways using Pearson correlation analysis (|r |> 0.9). Based on K-means clustering of the DVOC profiles, we further focus on 11 key volatiles that exhibited consistently increasing accumulation patterns from S2 to S4, mainly belonging to clusters 6 and 9. These included eugenol; 3-phenylpropanol; phenol, 4-(2-propenyl)-; benzyl alcohol, TMS derivative; acetic acid, phenylmethyl ester; acetic acid, cinnamyl ester; benzaldehyde; cinnamaldehyde, (E)-; octanal; geraniol; 5-hepten-2-one, 6-methyl-. A correlation network constructed from these 11 volatiles and 47 highly associated structural genes revealed potential links between floral scent compounds and genes (**Supplementary Figure S4**).

**Figure 7:**
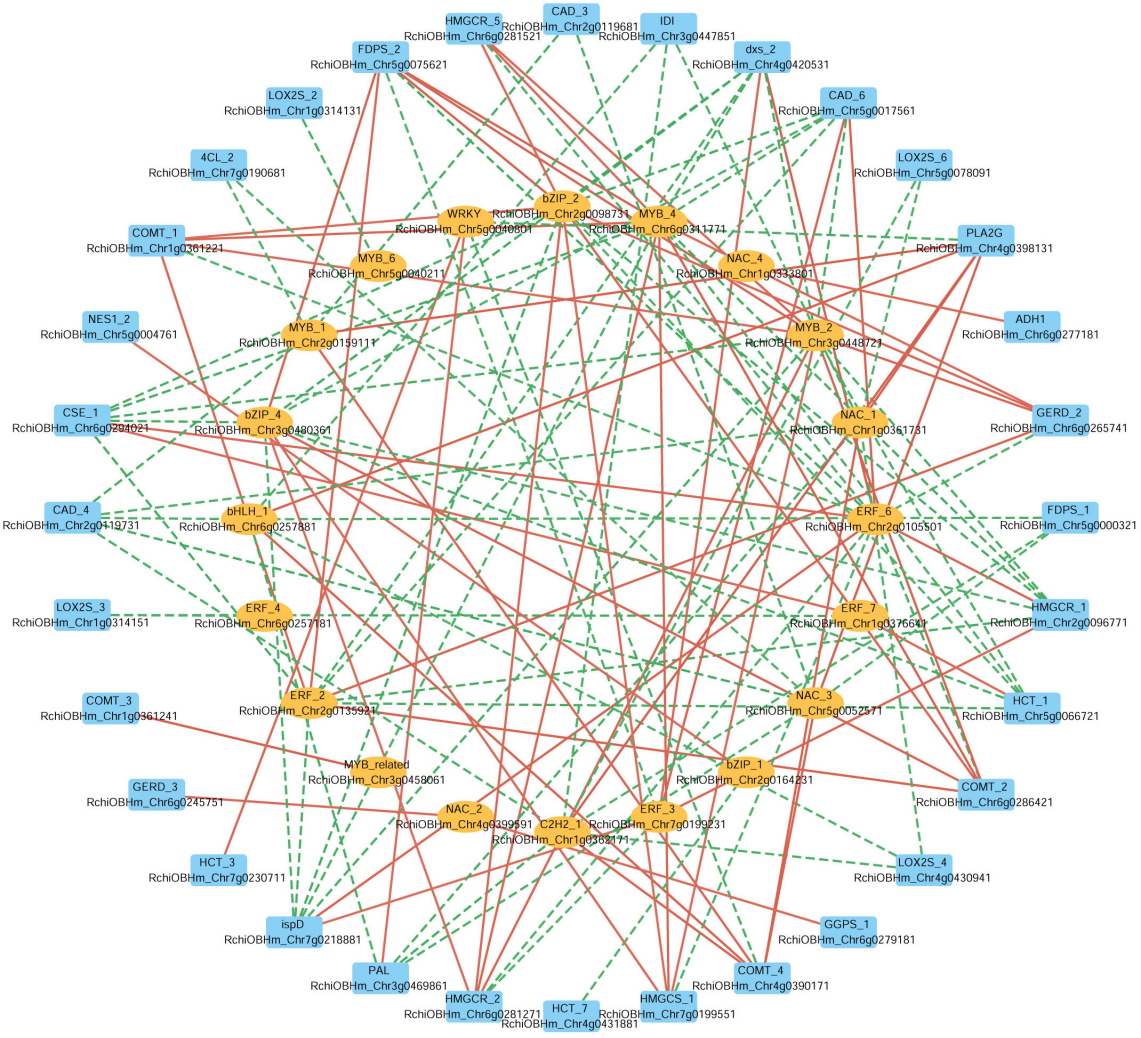
Correlation network between transcription factors and structural genes involved in phenylpropanoid biosynthesis, terpenoid biosynthesis, and fatty acid derivatives metabolism. The outer circle represents structural genes; the inner circle represents transcription factors; the color of edge: red indicates positive correlation, green indicates negative correlation.

In parallel, to further investigate the potential transcriptional regulation of these biosynthetic pathways, we selected 28 TFs from eight TF families (NAC, MYB, ERF, bHLH, C2H2, WRKY, bZIP, MYB_related), and analyzed their correlations with the same set of 67 structural genes. A Pearson correlation-based network (|r| > 0.9) (**Figure 7**) reveals 20 TFs significantly correlated with 32 structural genes, of which 18 TFs were positively correlated with 22 structural genes. For instance, in the phenylpropanoid biosynthesis pathway ERF_6 (RchiOBHm_Chr2g0105501) and ERF_3 (RchiOBHm_Chr7g0199231) were positively correlated with CAD_6 (RchiOBHm_Chr5g0017561). Additionally, ERF_7 (RchiOBHm_Chr1g0376641) and WRKY (RchiOBHm_Chr5g0040801) were positively correlated with HCT_1 (RchiOBHm_Chr5g0066721) and HCT_3 (RchiOBHm_Chr7g0230711), respectively. In the terpenoid biosynthesis pathway, bZIP_1 (RchiOBHm_Chr2g0164231) was positively correlated with NES1_2 (RchiOBHm_Chr5g0004761). In the fatty acid derivative metabolism pathway, NAC_4 (RchiOBHm_Chr1g0333801) was positively correlated with ADH1 (RchiOBHm_Chr6g0277181). These TFs might serve as potential regulators of the floral scent biosynthesis in *R. laevigata*.

### 3.8 qRT-PCR validation

**Figure 8:**
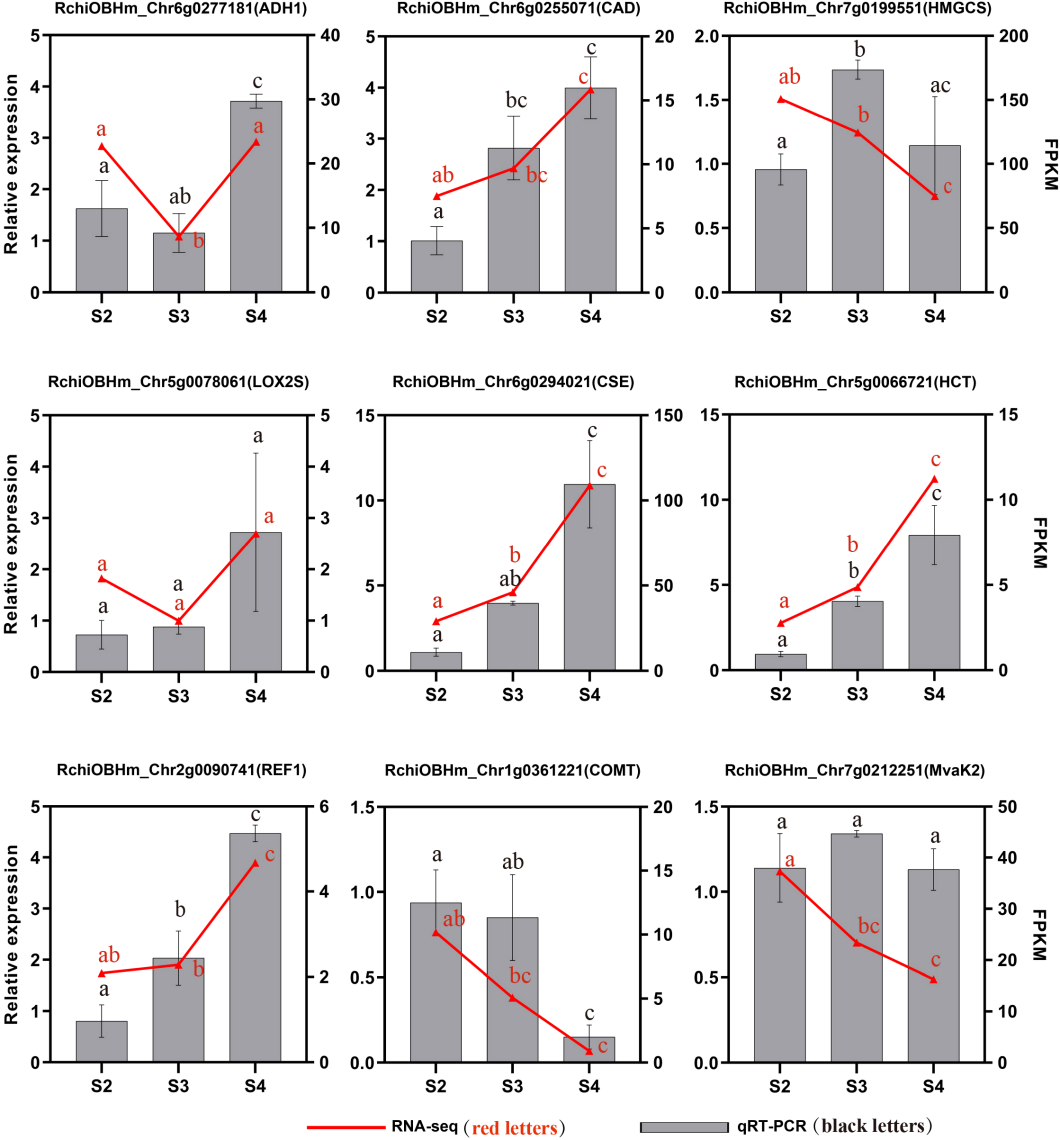
qRT-PCR validation of genes related to floral scent biosynthesis of *R. laevigata* flowers at three developmental stages. Different lowercase letters indicate significant differences among stages, as determined by one-way ANOVA (P < 0.05).

To validate the transcriptome data, we selected nine genes related to the phenylpropanoid biosynthesis pathway, terpenoid biosynthesis pathway, and fatty acid derivative metabolism pathway. Their expression levels in S2, S3, and S4 were analyzed using qRT-PCR. The relative expression levels of most candidate genes showed trends consistent with those observed in the transcriptome data analysis, confirming the reliability and reproducibility of the RNA-seq (**Figure 8**).

## 4 Discussion

### 4.1 VOCs of *R. laevigata* flowers

Floral scent is an important category of plant VOCs and a significant horticultural trait. It not only enhances the ornamental value of flowers but also influences the marketability of cut flowers (Sexton et al., 2005). To date, over 1,700 floral scent compounds have been identified, originating from 38 orders and 90 families (Knudsen et al., 2006). In this study, HS-SPME-GC-MS was used to analyze the VOCs of *R. laevigata* flowers at three developmental stages. The results showed that a total of 330 VOCs were identified in the flower samples, with 330 VOCs at S2 and 325 VOCs at both S3 and S4 (**Supplementary Table S2**). Previous studies by Wang(2019) and Jiao (2022) detected 10 and 31 VOCs at the fully blooming stage of *R. laevigata* petals and whole flowers, respectively. Our study identified VOCs that had not been previously reported in *R. laevigata* flowers, such as phenol, 3-methyl-, formic acid, octyl ester, acetic acid, cinnamyl ester, cinnamaldehyde, (E)-, etc. Therefore, this study significantly enriched the diversity of VOCs in *R. laevigata* flowers through metabolomics analysis, providing valuable data for the efficient utilization of fragrant *R. laevigata* germplasm.

The composition and relative abundance of floral scents vary greatly among different species or cultivars and are influenced by factors such as growth environment, developmental stage, collection time, and sampling location within the same species (Fan et al., 2019; Cheng et al., 2022; Jiao et al., 2022; Wang et al., 2022; Zhou et al., 2023a, b, 2024; Haghshenas et al., 2024). In this study, a total of 192 DVOCs were identified and clustered by K-means into four temporal trends based on their relative abundances from stages S2 to S4. These DVOCs were mainly classified into benzenoids/phenylpropanoids and ester, which were the most abundant classes, followed by terpenoids, heterocyclic compounds, and aldehydes. The DVOCs classes varied among these trends. The 102 DVOCs in clusters 1, 3, 4, and 5 exhibited a peak at stage S3, increasing from S2 and declining at S4. At S4, as floral organs expand and bloom, the accumulated volatiles are rapidly released, resulting in a decreased relative abundance within the tissues. This trend likely serves a dual function: providing strong olfactory cues to attract pollinators at the moment of flowering (Zhang et al., 2023), and forming a chemical barrier through volatile such as hexanoic acid, nonanal, dibutyl phthalate, caryophyllene oxide, which may inhibits pathogen infection and deters florivorous insects, thereby protecting immature floral organs (Scalschi et al., 2013; Zhang et al., 2017; Hilgers et al., 2021; Li et al., 2022). Clusters 2 and 8 showed the highest relative abundances at the S2 and were dominated by heterocyclic compounds and aldehydes. These volatiles may be associated with early-stage defense of floral organs against insect herbivores or microbial pathogens, as supported by previous findings on aldehydes (e.g., hexanal, 3-hexenal, (E)-4-oxohex-2-enal) (Scala et al., 2013) and monoterpenes (α-phellandrene, α-myrcene) (Zhang et al., 2017). The DVOCs in clusters 6 and 9 increase progressively in relative abundance from S2 to S4, reaching their highest levels at full bloom (S4). These clusters contain key constituents such as eugenol, geraniol, benzaldehyde and acetic acid phenylmethyl ester, all of which have been reported to play important roles in floral scent biosynthesis (Knudsen et al., 2006; Shi and Zhang, 2022; Dötterl and Gershenzon, 2023). These volatiles may collectively contribute to the complex and characteristic floral scent of *Rosa laevigata*.

### 4.2 Expression patterns of genes related to floral scent biosynthesis of *R. laevigata*

The formation of floral scent is a complex process that depends on the biosynthesis pathways of aromatic compounds and the regulation of their associated genes (Xiang et al., 2007; Bechen et al., 2022). In this study, 8,585 DEGs were identified in *R. laevigata* flowers at three developmental stages through transcriptome analysis. The number of DEGs increased progressively with flower development (**Figure 3B**). In each comparison group, the number of upregulated DEGs exceeded that of downregulated DEGs, suggesting that the production of VOCs at different stages of flower development may be linked to the activation or inhibition of these DEGs. KEGG enrichment analysis identified three key floral scent biosynthesis pathways and their associated genes.

The phenylpropanoid biosynthesis pathway is a rich source of plant secondary metabolites (Dong and Lin, 2021). In this study, 57 DEGs related to phenylpropanoid biosynthesis were identified from the transcriptome data, of which 30 DEGs were associated with floral scent biosynthesis. These genes encode nine enzymes, including PAL, 4CL, HCT, CCoAOMT, CCR, CAD, CSE, COMT, and REF1 (**Figure 5A**), which are essential for the biosynthesis and accumulation of benzenoids and phenylpropanoids (Wu et al., 2024). The biosynthesis of phenylpropanoids begins with phenylalanine, produced through the shikimate pathway, which is converted to cinnamic acid by phenylalanine ammonia-lyase (PAL) (Qiao et al., 2021). One gene, RchiOBHm_Chr3g0469861, encoding PAL, was identified with the highest expression at S2, gradually downregulating as flower development progressed (**Figure 5A**, **Supplementary Table S9**). Among the identified genes in this pathway, the largest number of genes encoded HCT, CAD, and COMT enzymes, though their expression patterns varied at different stages. Three genes, RchiOBHm_Chr5g0066721 (HCT), RchiOBHm_Chr2g0106691 (CAD), and RchiOBHm_Chr6g0255071 (CAD), showed continuously upregulated expression during flower development. Conversely, other genes exhibited continuously downregulated expression, including RchiOBHm_Chr7g0230501 (HCT), RchiOBHm_Chr7g0230711 (HCT), RchiOBHm_Chr1g0361221 (COMT), and RchiOBHm_Chr6g0286421 (COMT), or displayed mixed regulatory patterns. Notably, RchiOBHm_Chr5g0066721 (HCT), RchiOBHm_Chr6g0255071 (CAD) and RchiOBHm_Chr6g0294021 (CSE) showed positive correlations with eugenol, a key floral scent in the phenylpropanoid pathway (Wang et al., 1997; Koeduka et al., 2006), highlighting their potential roles in its biosynthesis.

Terpenoids are the largest class of natural floral scent compounds in plants. Their biosynthesis primarily originates from the precursor substances isopentenyl-PP or dimethylallyl-PP, through the mevalonate pathway (MVA) occurring in the cytoplasm and the methylerythritol phosphate pathway (MEP) occurring in the plastids (Vranová et al., 2013). Based on the transcriptome data, 26 DEGs were identified in the terpenoid biosynthesis pathway, with more DEGs found in the MVA pathway than in the MEP pathway (**Figure 6**). In the upstream MVA pathway, only one gene (RchiOBHm_Chr2g0096771) encoding HMGCR was upregulated from S2 to S4, while the remaining genes were highly expressed at S2 or S3 and were downregulated at S4. In the downstream MVA pathway, genes encoding AFS1 and NES1 related to sesquiterpenoid biosynthesis exhibited the same expression pattern, with the highest expression levels at S4 (**Figure 6**). However, genes encoding GERD enzyme showed varied expression patterns: continuously downregulated (RchiOBHm_Chr6g0265741, RchiOBHm_Chr6g0245751), continuously upregulated (RchiOBHm_Chr7g0212441), or high expression at S3 (RchiOBHm_Chr5g0038101). This variation suggests that different GERD homologs may have distinct functions and expression levels, leading to the production of different compounds. A similar expression pattern has been observed in Chrysanthemum indicum var. aromaticum for genes encoding GERD (Zhu et al., 2022). In the downstream MEP pathway, two genes (RchiOBHm_Chr6g0279181, RchiOBHm_Chr3g0493061) encoding GPPS and one gene (RchiOBHm_Chr3g0484891) encoding ispS showed similar expression patterns, with the highest expression at S2 and gradually downregulation as the flower developed. GPPS activity influences the synthesis of products in the isoprenoid pathway and plays an important regulatory role in the biosynthesis of monoterpenoids (Kamran et al., 2020).

Fatty acid derivatives are another class of VOCs that constitute floral scent, derived from the degradation of C18 fatty acids, such as linolenic and linoleic acids (Feussner and Wasternack, 2002). In this study, 11 genes encoding four enzymes (PLA2G, DAD1, LOX2S, ADH1) were identified in α-linolenic acid metabolism pathway, of which eight genes encode the main regulatory enzyme LOX2S. The expression levels of two genes encoding LOX2S (RchiOBHm_Chr5g0078061, RchiOBHm_Chr5g0078091) and one gene encoding ADH1 (RchiOBHm_Chr6g0277181) were downregulated from S2 to S3, then upregulated at S4. This trend was consistent with the relative abundance changes of VOCs such as 2-Penten-1-ol, acetate, (Z)-, and octanoic acid, ethyl ester.

The formation of floral scent in *R. laevigata* is regulated not only by structural genes related to phenylpropanoid, terpenoid, and fatty acid derivative biosynthesis pathways but also by TFs. In this study, we identified 20 TFs from eight TF families (MYB, NAC, bZIP, ERF, WRKY, bHLH, C2H2, MYB_related) involved in the regulation of these pathways, of which 18 TFs were positively correlated with 22 structural genes (**Figure 7**). These TFs have been shown to regulate VOCs synthesis in various plants (Ramya et al., 2017; Ding et al., 2020; Yang et al., 2021; Lu et al., 2023; Fu et al., 2024). In PAP1 transgenic Rosa hybrids, anthocyanin pigment1 (PAP1), a member of the MYB family, can upregulate the biosynthesis of phenylpropanoid and terpenoid aromatic compounds (Zvi et al., 2012). Zhou et al. (2023a) revealed that eugenol synthase and isoeugenol synthase are regulated by key TFs MYB and bHLH, influencing the floral scent of *Rosa yangii*. These results suggest that the coordinated regulation of TFs and DEGs plays a crucial role in the biosynthesis of VOCs, which are essential for attracting pollinators and reproductive strategies of plants. The identification of TFs provides a theoretical basis for further studies on their regulatory roles in the VOCs biosynthesis pathways of *R. laevigata*.

## 5 Conclusion

This study integrated metabolomic and transcriptomic analyses to investigate the molecular mechanisms of the VOCs formation in *R. laevigata* flowers. A total of 192 DVOCs were identified, among which benzenoids/phenylpropanoids and esters were the most abundant, potentially serving as the primary components of the floral scent. In the phenylpropanoid, terpenoid, and fatty acid derivative biosynthesis pathways related to floral scent formation, 67 DEGs were identified, showing significant changes in expression levels from the bud stage to full bloom, reflecting the complexity of regulatory network involved in floral scent biosynthesis. 20 TFs from the EFR, MYB, NAC, bZIP, bHLH, C2H2, MYB_related, and WRKY families were identified as potential regulators of genes involved in floral scent biosynthesis during the flower development. This study provides valuable data for the efficient utilization of fragrant *R. laevigata* germplasm, laying a foundation for the targeted breeding of floral scent and further promoting the application of *R. laevigata* in the aromatic flower industry.

## Data Availability

The data presented in the study are deposited in the CNGB Sequence Archive (CNSA) of the China National GeneBank DataBase (CNGBdb), accession number CNP0005764.
