# Supplementary material for: Molecular insights into floral scent biosynthesis in Rosa laevigata through transcriptomic and metabolomic analyses
Source: Front Plant Sci. 2025 Jun 23;16:1599758. doi: 10.3389/fpls.2025.1599758 (PMC12230050; doi:10.3389/fpls.2025.1599758)
Supplement: Supplementary file 1 [file DataSheet1.docx]

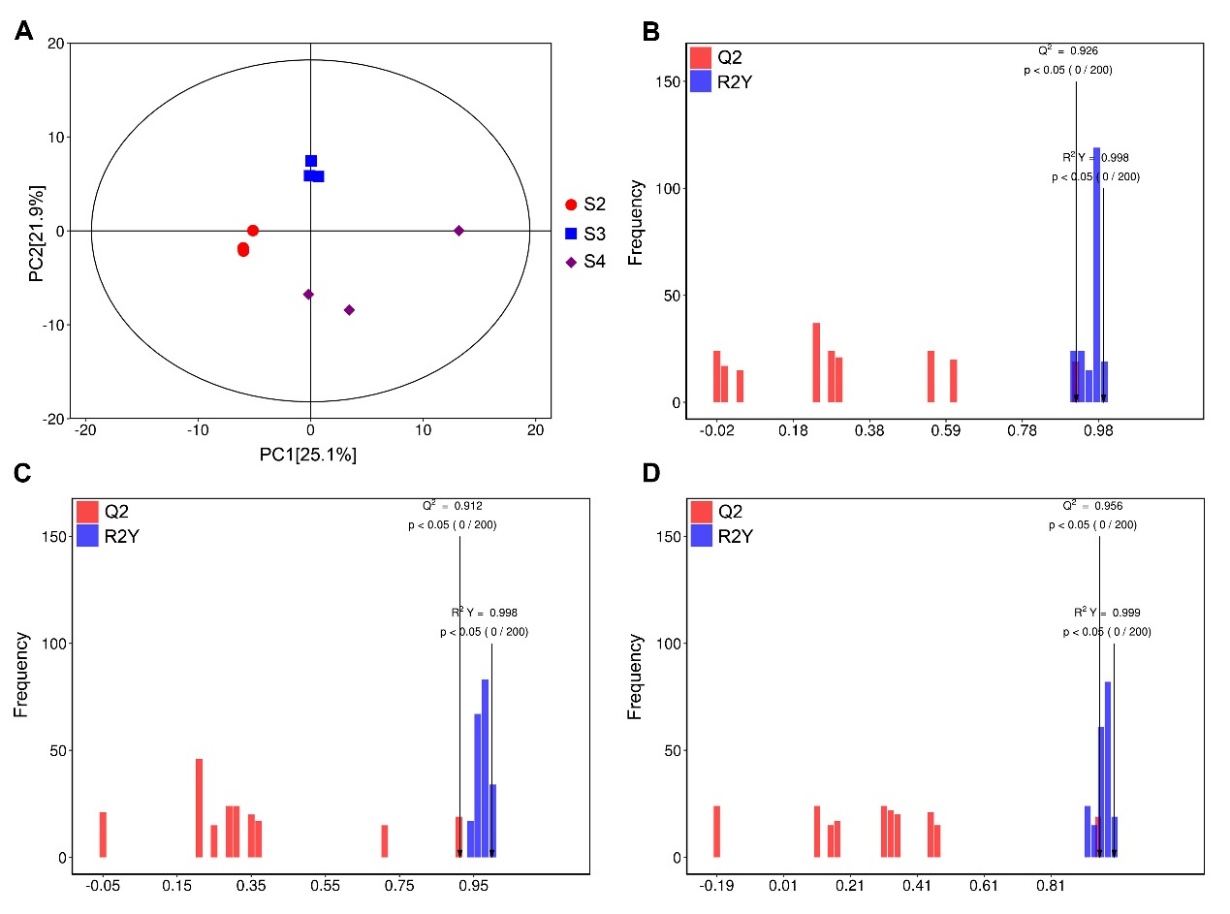


**Supplementary Figure 1.** Metabolome analysis of *Rosa laevigata* flowers at three developmental stages. **(A)** Principal component analysis of volatile organic compounds. **(B-D)** The permutation tests of the OPLS-DA models for S2 vs. S3 **(B)**, S3 vs. S4 **(C)**, and S2 vs. S4 **(D)**.


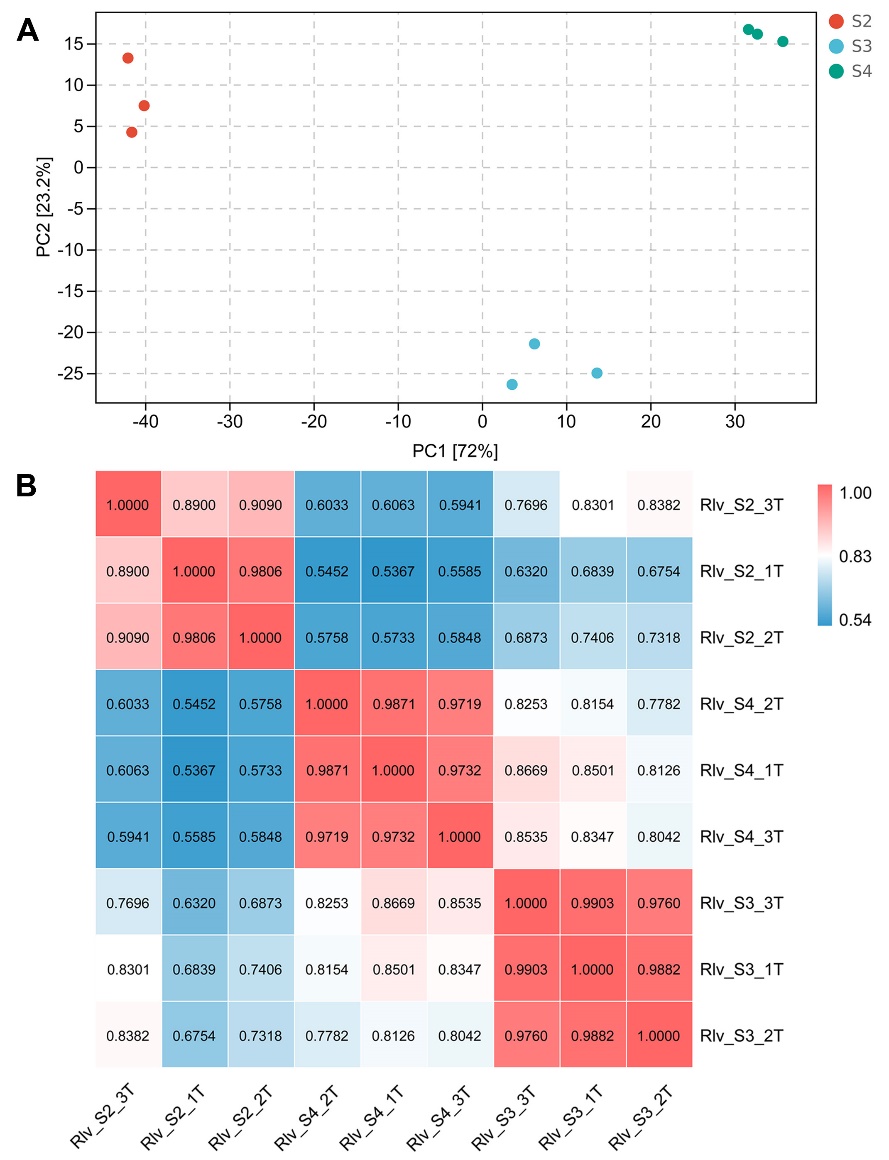


**Supplementary Figure 2.** Quality evaluation of transcriptome data of *Rosa laevigata* flowers at three developmental stages. **(A)** Principal component analysis. **(B)** Sample correlation analysis.


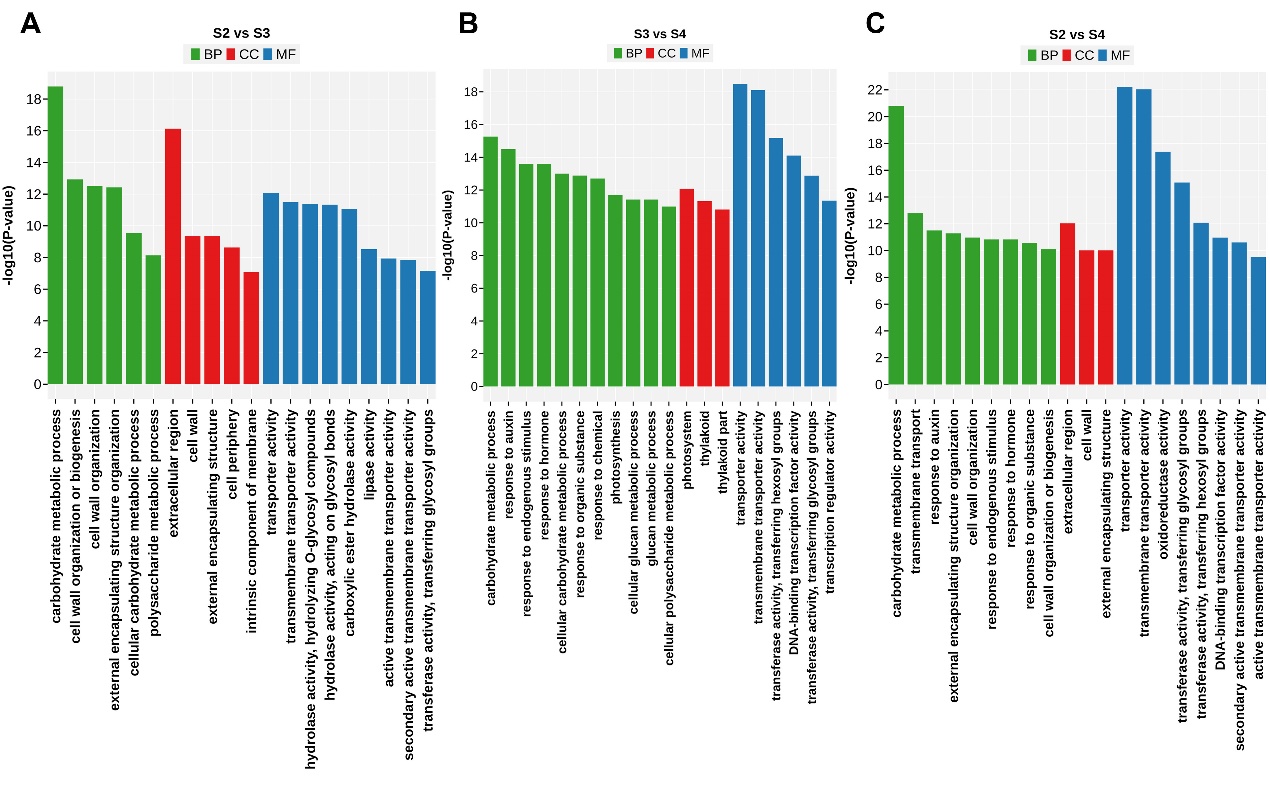


**Supplementary Figure 3.** The top 20 significantly enriched GO terms in the three comparison groups. **(A)** S2 vs. S3. **(B)** S3 vs. S4. **(C)** S2 vs. S4.


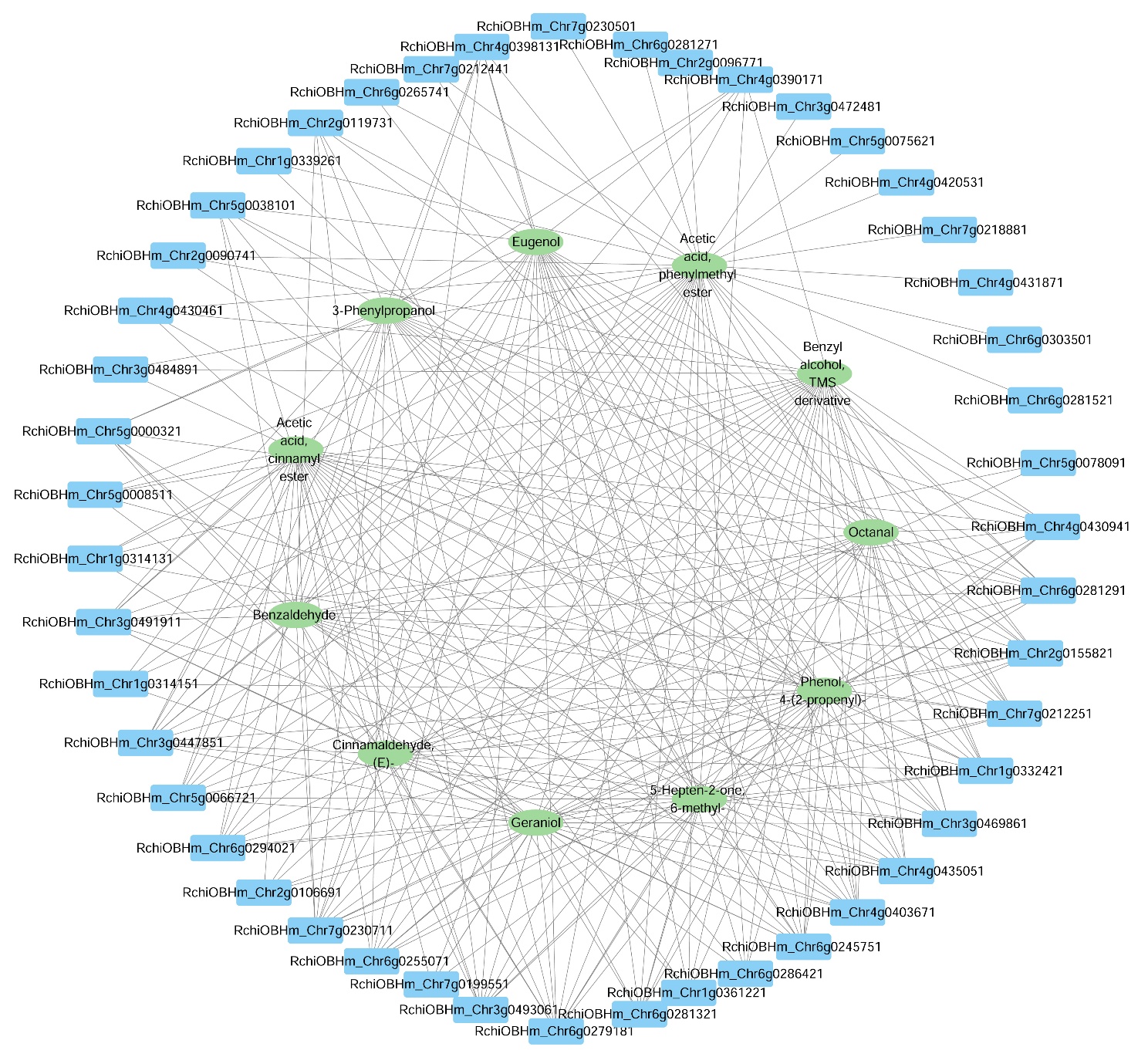


**Supplementary Figure 4.** Correlation network between floral volatile and structural genes involved in phenylpropanoid biosynthesis, terpenoid biosynthesis, and fatty acid derivatives metabolism. The outer circle represents structural genes, the inner circle represents floral volatile.
